# Correction to: Separate Gut Plasma Cell Populations Produce Auto‐Antibodies against Transglutaminase 2 and Transglutaminase 3 in Dermatitis Herpetiformis

**DOI:** 10.1002/advs.202400894

**Published:** 2024-03-13

**Authors:** S. Das, J. Stamnaes, E. Kemppainen, K. Hervonen, K. E. A. Lundin, N. Parmar, F. L. Jahnsen, J. Jahnsen, K. Lindfors, T. Salmi, R. Iversen, L. M. Sollid

*Adv Sci (Weinh)*. **2023** Sep;*10*(25):e2300401. https://doi.org/10.1002/advs.202300401. Epub 2023 Jul 9. PMID: 37424036

Description of errors:

1. Figure 1: In the original figure, the heading of Fig 1B, right panel, erroneously reads “Anti‐TG2 IgG”. The heading has now been changed corrected to “Anti‐TG3 IgG”.


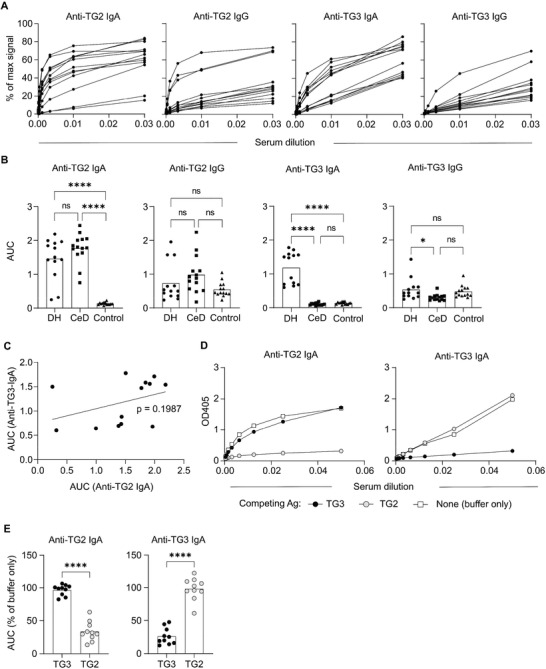


2. Figure 3: In the original figure, a legend explaining the symbols is missing in panel 3C. This legend has now been introduced.


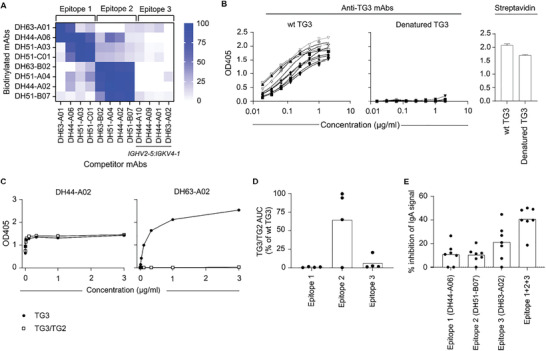


3. Table S1 (Supporting Information): In the original table, one monoclonal antibody (DH44‐A10) is missing. The information for this antibody has now been included.

We apologize for these errors.

On behalf of the authors,

Rasmus Iversen

Ludvig M Sollid

## Supporting information

Supporting Information

