# Supplementary material for: Correction to: Separate Gut Plasma Cell Populations Produce Auto‐Antibodies against Transglutaminase 2 and Transglutaminase 3 in Dermatitis Herpetiformis
Source: Adv Sci (Weinh). 2024 Mar 13;11(21):2400894. doi: 10.1002/advs.202400894 (PMC11151051; doi:10.1002/advs.202400894)
Supplement: Supplementary file 1 — Supporting Information [file ADVS-11-2400894-s001.pdf]

ADVANCED SCIENCE
Open Access

Supporting Information

for *Adv. Sci.*, DOI 10.1002/advs.202400894

Correction to: Separate Gut Plasma Cell Populations Produce Auto-Antibodies against Transglutaminase 2 and Transglutaminase 3 in Dermatitis Herpetiformis

S. Das, J. Stamnaes, E. Kemppainen, K. Hervonen, K. E. A. Lundin, N. Parmar, F. L. Jahnsen, J. Jahnsen, K. Lindfors, T. Salmi, R. Iversen and L. M. Sollid

Separate Gut Plasma Cell Populations Produce Auto-antibodies against Transglutaminase 2 and Transglutaminase 3 in Dermatitis Herpetiformis

Saykat Das, Jorunn Stamnaes, Esko Kemppainen, Kaisa Hervonen, Knut E.A. Lundin, Naveen Parmar, Frode L Jahnsen, Jørgen Jahnsen, Katri Lindfors, Teea Salmi, Rasmus Iversen and Ludvig M. Sollid**

*Email: rasmus.iversen@medisin.uio.no, l.m.sollid@medisin.uio.no

Supporting information

Supplementary Figures

- Figure S1. Gating strategy.
- Figure S2. TG2 and TG3 protein expression.
- Figure S3. V-gene usage by TG2-specific serum IgA in DH patients.

Supplementary Table

- Table S1. Sequences and sequence-properties of TG3-specific mAbs.

Reference

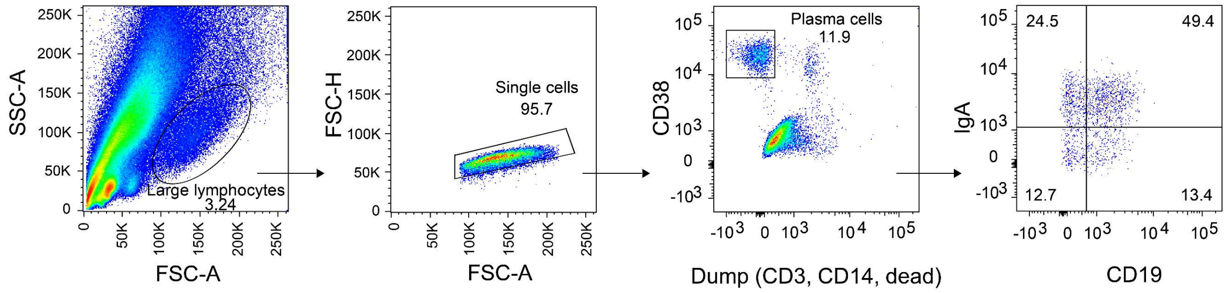

Figure S1. Gating strategy. Representative flow cytometry plots showing identification of IgA plasma cells in duodenal biopsy single-cell suspensions of DH patients.

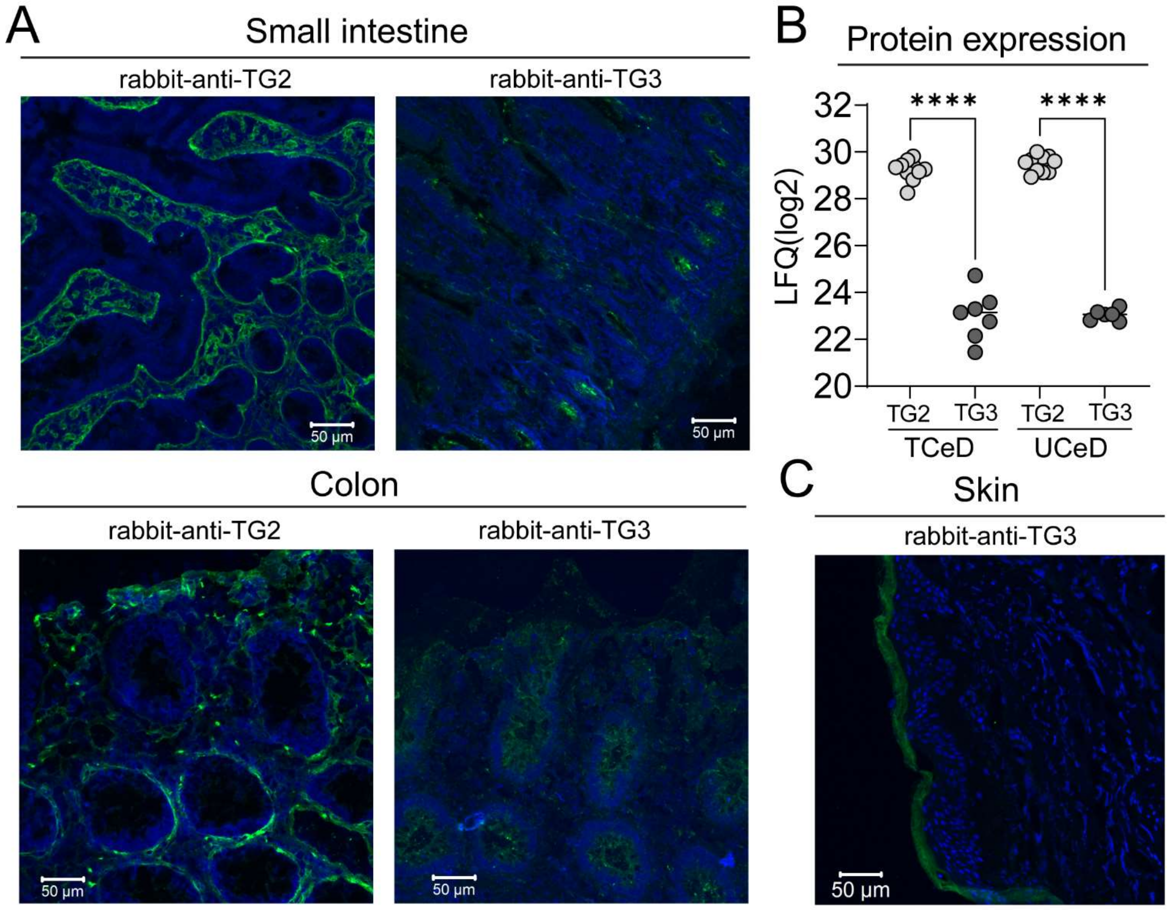

Figure S2. TG2 and TG3 protein expression. (A) Staining pattern for TG2 (polyclonal rabbit antihuman TG2 antibody) and TG3 (polyclonal rabbit antihuman TG3 antibody) in unfixed frozen sections from human small intestine and colon. Nuclei were stained with 4′,6-diamidino-2-phenylindole (DAPI). Scale bars; 50µm. (B) Comparison of TG2 and TG3 protein expression in human small intestine. The plot shows expression values from a previously published dataset of LC-MS/MS based proteome analysis of FFPE biopsy tissue sections from treated (TCeD) and untreated (UCeD) patients.[1] Proteins were quantified by label-free quantification (LFQ). Each

circle represents expression values from one patient biopsy block. Expression was compared using one-way ANOVA with Tukey's adjustment for multiple testing (****p<0.0001). (C) Immunofluorescence staining of human skin using polyclonal rabbit antihuman TG3 antibody (green). Positive TG3 staining is observed in stratum corneum of epidermis.

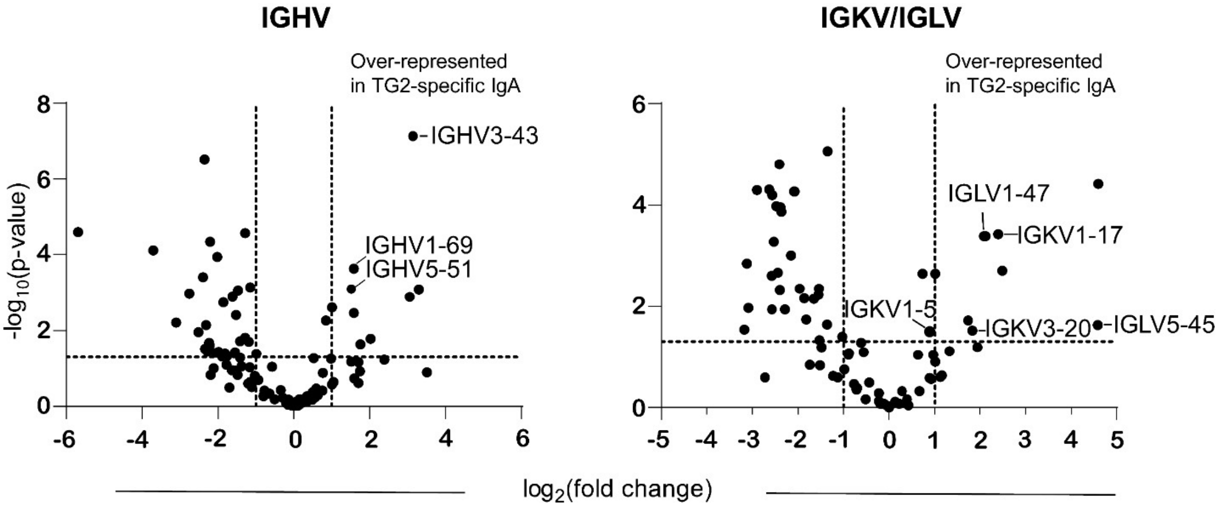

Figure S3. V-gene usage by TG2-specific serum IgA in DH patients. Volcano plots showing difference in *IGHV* and *IGKV/IGLV* usage between TG2-specific IgA and other IgA antibodies isolated from serum samples of DH patients (*n*=4). Statistical difference was evaluated by an unpaired t-test. Dashed lines indicate a p-value of 0.05 and a two-fold change in protein level based on label-free quantification (LFQ) intensity values obtained from MaxQuant. V-gene segments previously observed to be overrepresented among TG2-specific antibodies in CeD are indicated with their names.

Table S1. Sequences and sequence-properties of TG3-specific mAbs generated from gut plasma cells of DH patients

mAb ID	TG3 epitope group	Isotype	IGHV	IGHD	IGHJ	CDR-H3	CDR-H3 length	R	S	IGKV/IGLV	IGKJ/IGLJ	CDR-L3	CDR-L3 length	R	S	Clonality
DH63-A01	1	IgA1	IGHV3-48	IGHD2-21	IGHJ4	ASISSGNWYFDF	12	5	5	IGLV3-21	IGLJ2	QVWDTSDDLYVV	12	7	3	
DH51-A03	1	IgA1	IGHV3-30	IGHD2-21	IGHJ6	ARDVLTYSLYGMDV	14	21	10	IGKV3-15	IGKJ2	QMYDNWPPYT	10	2	77	
DH44-A06	1	IgA1	IGHV3-23	IGHD1-26	IGHJ4	AKDHGWELLTLYFDY	15	5	1	IGLV2-14	IGLJ2	TSYTTSSTPDVV	11	5	0	
DH51-C01	1	IgA1	IGHV3-23	IGHD3-9	IGHJ4	AKDQPGPPYYDILTGAFDY	19	6	1	IGLV2-23	IGLJ3	CSYAGSSTWV	10	2	1	
DH51-A04	2	IgA1	IGHV4-59	IGHD3-3	IGHJ3	ARDTHFGDAFDI	12	3	1	IGKV3-15	IGKJ3	QQYNYWPPPFT	11	7	3	
DH44-A02	2	IgA1	IGHV3-23	IGHD2-2	IGHJ4	AKDRSTLPPFDF	12	16	4	IGLV3-21	IGLJ3	QLWDSFSDARV	11	18	2	
DH51-B07	2	IgA1	IGHV1-8	IGHD2-2	IGHJ6	ATERNFCRDSSCSENSYMDV	21	6	3	IGKV3-11	IGKJ3	QQRSNWPPIFS	10	1	1	
DH63-B02	2	IgA1	IGHV3-9	IGHD3-10	IGHJ6	AKDHYLGSDSYGMDV	15	6	4	IGLV6-57	IGLJ2	QSYDPSNVV	9	6	0	
DH63-A02	3	IgA2	IGHV2-5	IGHD3-16	IGHJ4	AHRFVGTLDV	10	7	1	IGKV4-1	IGKJ1	QQYYSLWT	8	1	1	
DH44-A09	3	IgA1	IGHV2-5	IGHD3-16	IGHJ4	AHRRGGLTFDY	11	2	2	IGKV4-1	IGKJ1	QQYYSLWT	8	1	0	DH44-A01; DH44-A10
DH44-A01	3	IgA1	IGHV2-5	IGHD3-16	IGHJ4	AHRQGGLTFDY	11	3	0	IGKV4-1	IGKJ1	QQYYTLWT	8	0	0	DH44-A09; DH44-A10
DH44-A10	3	IgA2	IGHV2-5	IGHD3-16	IGHJ4	AHRRGGLTFDY	11	14	0	IGKV4-1	IGKJ1	QQYYTLWT	8	5	7	DH44-A09; DH44-A01

R: replacement mutations, S: silent mutations

Reference:

[1] A. E. V. Tutturen, S. Dørum, T. Clancy, H. M. Reims, A. Christophersen, K. E. A. Lundin, L. M. Sollid, G. A. de Souza, J. Stamnaes, *Am J Pathol* **2018**, *188* (7), 1563, https://doi.org/10.1016/j.ajpath.2018.03.017.
